# A. V. M. A.

**Published:** 1902-12

**Authors:** 


### A. V. M. A.

As we go to press the Secretary of the A. V. M. A. announces that some twelve of the fifteen votes of the Executive Committee have been cast in favor of Ottawa, Canada, thus insuring the success of that city. The strength of the profession in Canada should alone assure the success of the meeting, while the presence of the two schools at Montreal and Toronto, and the strong veterinary organization of Manitoba, are factors that must add much to the importance and influence in that territory for the future welfare of the profession.

Canada has long recognized the worth of the veterinary profession in governmental encouragement of the cause of higher veterinary education, the appointment of veterinary sanitary officers, and the government halls of legislation, all of which augurs well for the success of the meeting.

